# Physical Therapy to Prevent Osteopenia in Preterm Infants: A Systematic Review

**DOI:** 10.3390/children8080664

**Published:** 2021-07-30

**Authors:** Galaad Torró-Ferrero, Francisco Javier Fernández-Rego, Antonia Gómez-Conesa

**Affiliations:** 1 International School of Doctorate of the Univesity of Murcia (EIDUM), University of Murcia, 30100 Murcia, Spain; 2 Department of Physical Therapy, Faculty of Medicine, University of Murcia, 30100 Murcia, Spain; fjfernan@um.es; 3 Research Group Research Methods and Evaluation in Social Sciences, Mare Nostrum Campus of International Excellence, University of Murcia, 30100 Murcia, Spain; agomez@um.es

**Keywords:** tactile-kinesthesic stimulation, physical therapy modalities, metabolic bone diseases, neonatal intensive care units, premature infant, densitometry, biomarkers

## Abstract

Background: During the last trimester of pregnancy, about 80% of the infant’s calcium is incorporated, and for this reason, preterm infants have less bone mineralization compared to those born at term. The aim of the present systematic review was to identify, evaluate and summarize the studies that deal with the effect of physiotherapy modalities in the prevention and treatment of osteopenia in preterm infants. Methods: A comprehensive search (09/2019–02/2021) using PubMed, Web of Science, SCOPUS, ProQuest, SciELO, Latindex, ScienceDirect, PEDro and ClinicalTrials.gov was carried out. The following data were extracted: The number of participants, characteristics of the participants, design, characteristics of the intervention, outcome measures, time of evaluation and results. A non-quantitative synthesis of the extracted data was performed. The methodological quality and risk of bias were assessed using a PEDro scale and ROB-2 scale, respectively. Results: A total of 16 studies were analyzed, presenting a methodological quality that ranged from 3 to 8 points, and all showed some concerns regarding their risk of bias. Almost all studies (15/16) used passive mobilizations with joint pressure to prevent osteopenia, but they differed in the intensity and frequency of application. Conclusions: A daily exercise program of passive mobilizations with joint pressure, improves bone mineralization in preterm infants admitted to neonatal units.

## 1. Introduction

Osteopenia is one of the frequent pathologies in the preterm population [1], which is mainly defined by a deficiency in bone mineral availability [2]. Osteopenia is reported to occur in 16% to 40% of very low birth weight infants, and in 50% of extremely low birth weight infants [3,4,5]. This entity is of great interest to professionals working in neonatal care units, since the increasing survival rate of preterm infants, due to advances in neonatal care, is increasing their morbidity in this regard [1]. 

The early termination of a pregnancy causes a sudden interruption in the vertical supply of nutrients, from the mother to the fetus. Consequently, the entry of proteins and minerals, which is essential for the formation of the bone matrix and bones, is interrupted [6]. In fact, it is in the last trimester of pregnancy when 80% of the total calcium and phosphorus gains occur, and this is the period in which most of the mineralization takes place [7]. For this reason, children born prematurely have smaller bones and less bone mineralization compared to children born at term [8], and therefore present growth retardation [9].

Some of the first clinical consequences of this condition include rickets, fractures, impaired respiratory function, and poor growth [10]. In this sense, bone mineral density and osteopenia have been shown to correlate with gestational age, birth weight, and height [2].

In relation to the treatments for osteopenia of prematurity, some physical therapy modalities have shown good results, mainly through the use of passive mobilizations with gentle joint compression [11]. 

Given the importance of the topic, some systematic reviews have previously been carried out. We found a review by Stalnaker, et al. from 2016 [12], which includes articles published up to 2012, a Cochrane review by Schulzke, et al. from 2014 [13], which includes articles published up to March 2013, and an update by Eliakim, et al from 2017 [14], which includes articles up to 2016. 

Taking into account the consequences that osteopenia can have in preterm infants, the derived problems, and its high prevalence, we proposed to carry out an updated systematic review of the various physiotherapy modalities. Thus, the aim of this study was to perform a systematic review to identify, evaluate and summarize studies, and to assess their risk of bias and methodological quality assessment on the efficacy of physiotherapy modalities in the prevention and treatment of osteopenia in preterm infants.

## 2. Materials and Methods

### 2.1. Eligibility Criteria

Studies were selected when they met the following inclusion criteria: 

Study designs: Randomized clinical trials, written in Spanish, English, French, Portuguese or Italian.

Publication date: Anything published prior to and inclusive of February 2021.

Characteristics of participants: Studies whose sample is at least equal to or greater than 5 participants per group by the end of the study, and whose study population consist of preterm infants.

Type of treatments: Treatments that use physiotherapy modalities, excluding those that use drugs or other non-physiotherapeutic procedures.

Outcome measures: Studies that consider bone mineralization, bone formation, bone resorption, size, weight, head circumference, or the number of fractures as measurement variables.

### 2.2. Information Sources

#### 2.2.1. Electronic Baseline Data

The following specialized Health Science bibliographic databases were consulted: PubMed, Web of Science, SCOPUS, ProQuest, SciELO, Latindex, ScienceDirect and PEDro.

#### 2.2.2. Other Sources of Information

Studies in progress were identified through searches of ClinicalTrials.gov (www.clinicaltrials.gov). Electronic journals were also hand searched.

The search was conducted between the months of September 2019 and February 2020.

### 2.3. Search Strategy 

The following main Medical Subject Heading (MeSH) terms were used for the search: physical therapy modalities, metabolic bone diseases, preterm infant. The following entry terms were used: physical therapy, physiotherapy, exercise, assisted physical exercise, physical activity, osteopenia, bone formation, bone mineralization, bone strength, bone mineral density, preterm infants and very low birth weight.

Similar descriptors were combined using the operator “OR”, whilst the operator “AND” was used to combine intersecting concepts. 

As an example, the following search strategy was used in the PubMed: 

((Physical therapy) OR (Physical activity) OR (Physiotherapy) OR (Exercise) OR (Assisted physical exercise)) AND ((Osteopenia) OR (Bone Formation) OR (Bone mineralization) OR (Bone disease) OR (Bone strength) OR (Bone mineral density)) AND ((preterm infants) OR (Premature infants) OR (Very low birth weight)).

### 2.4. Selection Process

Once the search was complete and duplicates eliminated, the studies were selected after having read their abstracts and, in case of doubt, after reading the full article, to check that they met all the eligibility criteria. The eligibility assessment was carried out independently by two researchers from the team. Disagreements were resolved by consensus. When no agreement could be reached, a third author decided. 

### 2.5. Data Collection Process

One researcher from the team collected the data from each article, and another reviewer checked the data. Disagreements were resolved by consensus. When no agreement could be reached, a third author decided. If any information collected was unclear, the authors of the reports were contacted for further information. 

### 2.6. Data Items

From each included trial, the information was collected on the following items:

The number of participants in each group; the participants’ characteristics, together with the inclusion and exclusion criteria of the trial; the type of study; the type of intervention (including type, duration, and frequency, both in the control group (CG) and in the experimental group (EG)); measurement instruments used (urine biomarkers, serum biomarkers, ultrasound, DEXA), timing evaluation and results obtained; year of publication.

### 2.7. Study Risk of Bias Assessment

After selecting and reading the studies, the PEDro scale was administered to assess the methodological quality [15] and the Cochrane ROB-2 scale was used [16,17] to determine the risk of bias. Both scales provide different and poorly correlated information [18], and both scales were assessed following their instructions using an excel sheet that was previously prepared, outlining the items to be administered. Once the studies were read, assessments were performed by two researchers independently, and discrepancies were resolved by consensus. If no agreement could be reached, a third author decided.

## 3. Results

### 3.1. Study Selection

**Figure 1:**
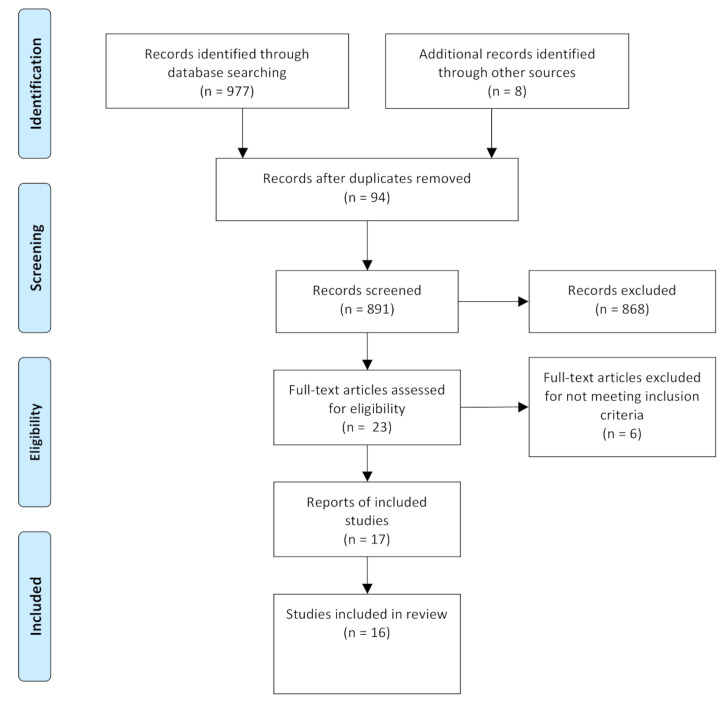
Flow diagram of the search results.

Through the literature search, 985 initial studies were identified (111 in PubMed, 76 in Web of Science, 73 in Scopus, 265 in ProQuest, 3 in Scielo, 0 in Latindex, 386 in ScienceDirect, 63 in PEDro and 8 in ClinicalTrials.gov). Of these, 94 were discarded because they were duplicates, and a further 868 papers were excluded when the researchers found that they did not meet the eligibility criteria, after reading the title and abstract. The remaining 23 full texts were reviewed. (Figure 1).

Ultimately, six articles were discarded after an in-depth reading because they did not meet some of the eligibility criteria: one of them did not study preterm infants, only their mothers [19]; another did not study undergoing physiotherapy treatment [20]; two studies did not measure bone variables [21,22]; one of the six studies in February 2020 is still pending completion and publication [23]; finally, one was excluded because the allocation was not randomized [24]. After the screening and eligibility process, we included 17 articles that led to 16 studies.

### 3.2. Study Characteristics

All studies included in the review were randomized clinical trials, published in English (Table 1). The 17 articles [11,25,26,27,28,29,30,31,32,33,34,35,36,37,38,39,40] that met all the eligibility criteria were analyzed. Since the articles by Vignochi, et al, 2008 and 2012 [31,32] belong to the same study, their characteristics were analyzed together.

### 3.3. Participants 

The total population analyzed of all the studies was 489, of which 258 (130 boys and 128 girls) belong to the experimental group (EG), and 231 (126 boys and 105 girls), to the control group (CG). Two of the studies, by Litmanovitz, et al. (2016) [39] and Moyer-Mileur, et al. (2008) [26] included two experimental groups. In the studies, no statistically significant differences were found between sex, gestational age, weight or height at baseline, or in the baseline measurements of the main variables. Gestational ages ranged from 26 to 32 wGA, birth weight from 900 g to 1900 g, and height from 30 cm to 43 cm.

In all the studies, as inclusion criteria, the authors considered that the infants were clinically stable. In ten, they specified that patients must present complete enteral nutrition (100–120 Kcal/kg/day intake) [11,25,26,27,30,31,32,35,36,37,40] and five specified a similar nutritional contribution among all participants [29,33,34,38,39].

Likewise, all the studies required that the participants had no health complications (Table 1).

### 3.4. Characteristics of the Physical Therapy Treatment 

As shown in Table 2, in 15 of the 16 studies [11,25,26,27,28,29,30,31,32,33,34,35,36,38,39,40], passive mobilizations with gentle joint compression (PMC), described by Moyer-Mileur, et al. (1995) [11], were used as a treatment modality, and one of the studies also used massage techniques [25]. Haley, et al. (2012) carried out a different protocol than the rest, with tactile and kinesthetic stimulation (TKs) [37]. This stimulation consists of passive mobilizations of the same joints described by Moyer-Mileur, et al. (1995) [11], but nothing indicates that they applied joint pressure [37].

Regarding the procedure for the CG, there are more differences. In nine studies tactile stimulation was used [11,26,27,33,34,35,38,39,40]. In another six the prescribed nursing care alone was administered to the CG [28,29,30,31,32,36,37], and one of the studies does not specify whether or not the CG carried out any activity [25]. In relation to the periods of application of treatment, in nine studies it was carried out for 4 weeks [11,27,30,33,34,35,36,38,39], 2 weeks in one [37], and another performed the treatments for 30 days [28]. In one study, they prolonged the treatment until hospital discharge [31], in another study the intervention was performed until reaching 40 wGA [29], and in three the weight was used as a criterion, either up to 2 kg [26,40] or up to 1.8 kg [25]. In 11 studies [25,29,30,31,33,34,35,36,38], 5 weekly treatment sessions were applied, ranging from 5 to 10 min, except for the study by Vignochi, et al., in which they increased the treatment times to 15 min [31,32]. However, Haley, et al. performed 12 weekly 20 min sessions [37], Sezer Efe, et al. [28] carried out 7 weekly sessions for a maximum of 10 min in duration, and Moyer-Mileur, et al. applied 6 weekly sessions for a maximum of 10 min [11,26,40]. In one of the studies, two experimental groups were included. For one they applied 10 weekly sessions and to the other 5 [39]. Finally, it should be noted that in all the studies, the treatments were applied at the hospital, except in the study carried out by Shaw, et al. [29], where treatment started at the hospital and was finished at home by the mothers. Most studies do not specify who was responsible for carrying out the treatment.

### 3.5. Outcome Measures

In the studies analyzed, regarding the evaluation moments, it was observed that in all of the measurements are made at the beginning and at the end of the treatments. In addition, in the studies of Litmanovitz, et al. [39] and Chen, et al. [33] intermediate measurements were included. Meanwhile, the trial by Chen, et al. [33] was the only one in which follow-up measurements were performed (Table 2).

Out of all the measurements, 12 of them (75%) used plasma biomarkers, 6 (37.5%) used urine biomarkers, 8 (50%) used ultrasound, 5 (31.25%) used bone densitometry, and in 14 (87.5%) anthropometric measurements were compared. All studies analyzed weight, and 11 (68.75%) analyzed height and head circumference (Table 2).

The markers of bone formation identified in the literature included bone-specific alkaline phosphatase (BSAP), osteocalcin and procollagen type I carboxy-terminal propeptide (PICP), parathyroid hormone (PTH), leptin levels, insulin growth factor type I (IGF-I), alkaline phosphatase (ALP), blood phosphorus (PO4), urinary osteocalcin medium fragment (U-MidOC), and cortisol levels. The markers of bone resorption included collagen carboxy-terminal cross-linked telopeptides type I (ICTP), pyridinoline cross-links (pyridinoline (Pyd) and deoxypyridinoline (Dpd)), urine calcium to phosphorus ratio (Ca/PO4), and carboxy-crosslinked telopeptide-terminal collagen type 1 (CTX). Improvements in the rate of bone formation were determined by an increase in the markers of bone formation and a decrease in the markers of bone resorption [12].

### 3.6. Risk of Bias in Studies

To assess the studies’ risks of bias, the PEDro scale [15] and the Cochrane ROB-2 scale [16,17] were applied. The results are shown in Table 3 and Table 4 respectively.

According to the PEDro scale, the lowest scores were 3, 4 and 5 points. A score of 6 was obtained in six of the sixteen studies analyzed, a score of 7 in four of them, and a score of 8 in the remaining two (since the first item was not taken into account in this score). Items 2 and 10 were the only ones that were met by all studies. Conversely, Items 6 and 9 were not met by any study.

According to the Cochrane ROB-2 scale, all the studies showed a low risk of bias regarding missing outcome data (100%), and measurement of the outcome (100%). In addition, 56.25% of the studies showed a low risk of bias in the randomization process, and only 12.5% showed a low risk of bias regarding the selection of the reported result. In terms of the deviations from the intended interventions, 100% showed some concerns of bias, none of the studies considered showed a high risk of bias in any domain, and also none of them showed a low risk of bias in all the items; for these reasons, all studies are considered as overall presenting some concerns of bias (100%).

Regarding the results obtained with the Cochrane ROB-2 scale, as these are non-pharmacological treatments, the nature of the interventions and the population to which they were applied—the fact that the participants and the therapists were not blinded—leads to “some concerns” about the risk of bias rather than a high risk of bias.

On the other hand, given that the assessment instruments were bone biomarkers, in the studies by Aly, et al. [25], Chen, et al. [33], Eliakim, et al. [35], Moyer-Mileur, et al. [11] Nemet, et al. [27] and Vignochi, et al. [31], despite not presenting assessor blinding, a high risk of bias could not be considered, since a subjective interpretation by the assessor is more difficult.

The studies by Chen, et al. [33], Eliakim, et al. [35], Erdem, et al. [36], Litmanovitz, et al. [38], Nemet, et al. [27] and Tosun, et al. [30], do not specify how random sequence generation and allocation concealment were performed. Therefore, due to the lack of sufficient information, “some concerns” about the risk of bias were determined.

Finally, as the previous protocols of the studies are not published (except those of El-Farrash, et al. [34] and Sezer Efe, et al. [28]), it is unknown if there are selective reports, so there are “some concerns” about the risk of bias. For both studies, a low risk of bias was determined in this regard, since they do not present selective reports, and they analyzed and published all previously established variables and measures.

With the administration of the Cochrane ROB-2 scale, all articles globally present an unclear risk of bias.

### 3.7. Results of Individual Studies

Among the authors that use serum biomarkers, Aly, et al. [25] found differences in favor of EG in the PICP measurements, but not of ALP or PTH; on the other hand, Nemet, et al. [27] found differences in the biomarkers of BSAP and ICTP, but not in those of PICP; Moyer-Mileur, et al. [11] found differences in ALP biomarkers in favor of GC and no differences were found in the rest of the biomarkers; Vignochi, et al. [32] found differences in the biomarkers of BSAP, but not in those of Ca, PO4 and PTH. Regarding the rest of the authors, Chen, et al. [33], El-Farrash, et al. [34], Eliakim, et al. [35], Litmanovitz, et al. [38], and Moyer-Mileur, et al. [26,40] found significant differences in favor of the group treated with PMC in the serum biomarkers they used. In contrast, Sezer Efe, et al. [28] did not find significant differences between their treatment groups in this regard.

Regarding urine biomarkers, Moyer-Mileur, et al. [11] found no differences between the groups, Aly, et al. [25] and Moyer-Mileur, et al. [31,40], did not find differences in Pyd values. El-Farrash, et al. [34] did not find differences between groups with respect to the values provided by the CTX test, but in the Ca/PO4 test in favor of the group treated with PMC. Vignochi, et al. [32] found differences in favor of the group treated with PMC in the Dpd test, and Haley, et al. [37], did not find differences in the urine biomarkers Pyd and Dpd, but in the levels of U-MidOC were in favor of the group treated with TKs.

Babies in the experimental groups treated with PMC and TKs showed a significant increase in 9 of the 12 studies (75%) that used biomarkers of bone formation and a significant decrease in only 4 of the 9 (44.44%) that used biomarkers of resorption.

As measurements of bone mineralization, US has been used for the measurement of tibial speed of sound, and densitometry with DEXA. Of the 8 studies that use US as a measure of bone mineralization, only one of them, Shaw, et al. [29], found no differences between treatment with PMC versus CG, while in the rest of the studies, Chen, et al. [33], Erdem, et al. [36], Litmanovitz, et al. [38], Efe, et al. [28] and Tosun, et al. [30], all found differences, with the group treated with PMC presenting better mineralization. Likewise, Litmanovitz, et al. [39], found that the differences increase with the increase of the frequency of treatment with PMC to 10 weekly sessions. Haley et al. [37] found differences in favor of treatment with TKs in relation to the measurement of tibial sound velocity. On the other hand, all the studies that use bone densitometry (Moyer-Mileur, et al. [11], El-Farrash, et al. [34], Moyer-Mileur, et al. [26,40] and Vignochi, et al. [31]), found significant differences between the group treated with PMC versus the CG.

Babies belonging to the physical activity groups showed favorable differences in relation to the tibial speed of sound in seven of the eight studies (87.5%), and in the five in which bone density measurements were carried out (100%).

Finally, in terms of anthropometric measures, we found disparity, since 8 of the 14 studies in which it was assessed (57.14%) found differences in weight in favor of the group that received physical activity (Moyer-Mileur, et al. [11,40], Vignochi, et al. [31], El-Farrash, et al. [34], Eliakim, et al. [35], Erdem, et al. [36], Litmanovitz, et al. [38], and Nemet, et al. [27]); and of the 11 that have assessed height and head circumference, 2 (18.18%) and 1 (9.09%) found differences in height and head circumference respectively in favor of exercise (Table 2).

## 4. Discussion

### 4.1. Summary of Evidence 

In relation to the characteristics of the sample, all the studies coincide in terms of the inclusion and exclusion criteria used. As we mentioned in the previous section, in a study (Efe, et al. [28]), it was not specified that if the nutritional intake was similar between groups, and treatments were started without having achieved complete enteral nutrition. This is an aspect that should be taken into account, since the amount of calories and minerals that these subjects ingest could affect the diagnosis and evolution of osteopenia.

In most studies, passive mobilizations with gentle joint compression described by Moyer-Mileur, et al. [11] were used, but they differed in terms of use of a placebo for the control group, the periods of application, and in the frequency and intensity of the treatment. The treatment with gentle joint compression has been suitable to encourage bone formation and mineralization in other studies [41,42]. The administration of a placebo treatment in the CG seems to be useful to eliminate biases and to eliminate the possible effect of any type of special care on infants who spend so many hours alone in the incubator.

Regarding frequency and intensity, according to the results of some studies, the higher they both are, the better the results obtained [31,32,39]. Still, larger populations and more homogeneous characteristics, especially with regard to gestational age, are recommended aspects for subsequent studies to consolidate these results.

Regarding the periods of the application of treatment, the studies in which it was administered up until hospital discharge, were equal to the 4 weeks that are carried out in other studies. Only the study by Shaw, et al. [29] differs; in this study the treatment lasted almost twice as long, and was applied up to 40 wGA. Nevertheless, no differences were found between the groups in terms of US measurements.

The outcome measurement are where the studies differed the most. There is a significant variation in the protocols for the diagnosis of osteopenia. A combination of different tests (bone biomarkers, densitometry or ultrasound), is often used to diagnose osteopenia of prematurity, but there is a lack of consensus on which screening tests and which thresholds to use [43]. This is largely due to a lack of normative data and clinical trials in preterm infants [43]. The fact that the studies analyzed used different outcome measures could make it difficult to generalize their results. Still, the fact that different studies using the same treatment techniques, but different outcome variables, reached similar conclusions, facilitates the study conclusions in this regard.

Most studies used serum biomarkers and anthropometric measurements, and although, in general, they all obtained significant results in favor of treatment with physiotherapy modalities, no significant differences were found with some serum and urine biomarkers, which could be due to the fact that the most appropriate ones had not been used. For further research, in terms of bone formation biomarkers, the use of osteocalcin markers and PICP would be more convenient, since they have been shown to be the most sensitive markers of bone formation [44]. Therefore, its use is recommended for future research. Bone alkaline phosphatase, on the other hand, is not an advisable biomarker, since it can be affected by the placental isoenzyme [45].

Bone resorption biomarkers are also convenient, since as Beyers, et al. [46] demonstrated in a study on postnatal bone mineralization, the osteopenia observed in preterm infants is mainly caused by increased bone resorption and less so by decreased bone formation [46]. Furthermore, as most of the resorption markers are urine, invasive interventions are avoided in the preterm infant, for whom painful procedures are highly harmful [47,48]. Therefore, it is convenient to use these tests to measure this variable due to its importance and impact.

Studies that used bone densitometry to establish the diagnosis could be more reliable, as they are considered to be the gold standard for the diagnosis of bone mineralization [49]. However, this should not undervalue the use of quantitative ultrasound devices; as they have been shown to be effective in assessing the state of the bones of preterm infants [50], and since this technique does not emit radiation, it is a more suitable device to use on this kind of population [51]. Studies in which PMC and bone densitometry or quantitative ultrasound were used as comparison measures, obtained favorable treatment results, with the exception of study by Shaw, et al. [29].

It is important to highlight that, although Haley, et al. [37] obtained good results measured with US, they did not observe significant differences in bone biomarkers. The studies that obtained the best results were those in which PMC was used in their intervention model. Shaw, et al. [29] did not find differences in US measurements, despite the fact that they carried out the intervention following similar procedures to the other authors. Two differences from most studies come to attention: firstly, the people in charge of carrying out the treatment were not expert professionals, but rather the mothers of the babies themselves, and it could be that they did not learn the procedure correctly, or that they did not perform the exercises at the established frequency, despite the great efforts carried out by the authors of the study to ensure that this was the case. Secondly, there is a difference in the treatments’ times of application, and thus also in the time elapsed until the last measurement. In this sense, this is the only study that extends the intervention by two months and only provides the average performed at the end of the treatment. These results could be explained by certain factors, such as the person applying the treatment, the duration of the treatment or the moment of measurement. However, the results contrast with those obtained in the studies by Aly, et al. [25] and Moyer-Mileur, et al. [26], which did find differences in the treatment when applied by mothers, and with the results obtained by Chen, et al. [33], who also found differences when performing follow-up measurements after 8 weeks with favorable results. Nevertheless, it is true that the study by Shaw, et al. [29] (which did not find these differences in favor of the experimental group) is the one with the best methodological quality, according to the PEDro scale, with respect to the last three.

Regarding the anthropometric measurements, it seems that these do not correspond to the favorable results observed in other variables, and there does not seem to be a clear link between the gain in weight, height, head circumference and bone mineralization. Only 8 of the studies that, among the anthropometric measurements, analyzed weight, found differences jointly with the variables of bone mineralization. It seems that this result could be due to a placebo effect, since tactile stimulation and massage applied to the control group, improved weight gain and growth in preterm infants [52,53].

All studies reached very similar conclusions in favor of the effect of PMC in improving bone mineralization in preterm infants, but the analyzed studies differ in terms of their methodological quality, and it is observed that the studies with better methodological quality and lower risk of bias coincide with the results obtained by the lower quality studies.

It is worth highlighting the fact that Moyer-Mileur, et al. [11], Aly, et al. 2004 [25], Chen, et al. [33], Eliakim, et al. [35], Nemet, et al. [27], and Vignochi, et al. [31], did not have assessor blinding. This qualitative aspect is not recommended, even when it comes to biochemical tests, where subjective interpretation by the evaluator is not possible.

One of the strengths of this systematic review, compared with previously published reviews, is the large number of relevant clinical trials (16) that it included. We employed a very thorough search strategy in order to identify most of the current evidence on the administration of physical therapy in preterm infants to prevent osteopenia. Nevertheless, it is possible that some studies may have only been published in local databases and were not, therefore, included in this review. Furthermore, we adhered to the PRISMA protocol [54] in order to achieve the most rigorous and explicit scientific design possible and facilitate reproducibility. Of all the reviews published previously on the subject under study, none of them follow the PRISMA recommendations for systematic reviews, and only the one published by Schulzke, et al. [13] did a risk of bias assessment, but they did not use a risk of bias assessment scale. The use of the PEDro scale and the ROB-2 scale for this review provided valuable quantitative information about the methodological quality and the risk of bias of the studies examined. The PEDro scale showed that 13 of the 16 studies present a score of 5 or higher and the ROB-2 scale showed that all studies present some concerns of bias, however, none of them present a high risk of bias for any item nor a low risk of bias for all items. The use of these scales in future reviews, or similar studies would facilitate the identification of progress in scientific evidence concerning the administration of physical therapy modalities in preterm infants.

We consider that another of the strengths of this review is that it considered a major part of the randomized clinical trials published over the past 25 years, thus presenting an updated synthesis of the scientific literature on the administration of passive mobilizations to prevent osteopenia in preterm infants. 

### 4.2. Limitations

One of the most important limitations observed in the studies is related to the evaluation times. In almost all of them, two evaluations were carried out, one prior to the treatment and another at the end of it. It would be convenient to carry out follow-up evaluations, some time after having finished it, in order to learn whether the results were maintained over time or only while the treatment was being carried out. Likewise, conducting intermediate evaluations can offer information about when changes occur. 

The main limitation of our study could be due to the loss of some articles in the search process, due to the limits of the search in terms of language and outcome measures. 

### 4.3. Implications for Clinical Practice

From the field of physiotherapy, two procedures have been used to address osteopenia—passive mobilizations with joint pressure and tactile and kinesthetic stimulation.

The results of this review show that the best physical therapy modality, and the one most used for the management of osteopenia, is passive mobilization with joint pressure.

Regarding the periods and times of application of treatment, despite the discrepancies found, the most appropriate treatment should last one month, carrying out passive mobilizations with gentle joint compression, as described by Moyer-Mileur [11], for five to six days per week, with sessions lasting from 10 to 15 min, and being able to carry out up to two daily sessions, with a total of 5 to 10 weekly sessions.

### 4.4. Implications for Future Research

For future research, it would be advisable to check if other physiotherapy modalities are effective in the treatment of osteopenia. Some of the studies included in this review and with good methodological quality in their procedures did not find favorable results in terms of bone mineralization using PMC [29].

It would also be advisable to carry out randomized clinical studies with a larger population of preterm infants and with greater homogeneity in some variables, such as those referring to nutrition and gestational age.

To more accurately determine when changes occur, and if these are maintained in the medium and long term, measures must be taken during treatment and some time after its end.

## 5. Conclusions

The data obtained in this review seem to indicate that a daily exercise program of passive mobilizations with gentle joint pressure, between 10 and 15 min a day, for 4 to 8 weeks, is the intervention that shows the best effects on improving the bone mineralization measured with US, densitometry, and biomarkers of bone formation and resorption, in preterm infants with adequate weight for their gestational age admitted to neonatal units

## 6. Other Information

### Protocol and Registration

We developed a review protocol in accordance with PRISMA (Preferred Reporting Items for Systematic Reviews and Meta- Analyses) guidelines [54]. The protocol can be found at PROSPERO. PROSPERO ID: CRD42020175149. Registered 18/05/2020; URL: https://www.crd.york.ac.uk/PROSPERO/display_record.php?RecordID=175149.

## Figures and Tables

**Table 1:** Study and participant characteristics.

Study	Participant Characteristics					Design	Study Characteristics	
	N		Details					
	EG	CG	wGA	Weight (gr)	Feeding		PEDro	Rob-2 Cochrane
Aly, et al. 2004 [25]	15	15	<35	NS	CEN	RCT	3/10	*Some concerns*
Chen, et al. 2010 [33]	8	8	<37	<1500	SN	RCT	5/10	*Some concerns*
El-Farrash, et al. 2019 [34]	18	18	≤32	≤1500	SN	RCT	6/10	*Some concerns*
Eliakim, et al. 2002 [35]	10	10	<37	<1500	CEN	RCT	4/10	*Some concerns*
Erdem, et al. 2015 [36]	14	14	≤32	<1000	CEN	RCT	7/10	*Some concerns*
Haley, et al. 2012 [37]	20	20	<33	NS	CEN	RCT	7/10	*Some concerns*
Litmanovitz, et al. 2003 [38]	12	12	<37	<1500	SN	RCT	6/10	*Some concerns*
Litmanovitz, et al. 2016 [39]	EG1: 14EG2: 11	10	<37	<1500	SN	RCT	6/10	*Some concerns*
Moyer-Mileur, et al. 1995 [11]	13	13	<34	NS	CEN	RCT	4/10	*Some concerns*
Moyer-Mileur, et al. 2000 [40]	16	16	<32	<1600	CEN	RCT	8/10	*Some concerns*
Moyer-Mileur, et al. 2008 [26]	EG1: 11EG2: 11	11	<32	<1600	CEN	RCT	6/10	*Some concerns*
Nemet, et al. 2002 [27]	12	12	<37	NS	CEN	RCT	6/10	*Some concerns*
Sezer Efe, et al. 2019 [28]	12	12	<32	<1500	NS	RCT	8/10	*Some concerns*
Shaw, et al. 2017 [29]	26	24	<35	NS	SN	RCT	7/10	*Some concerns*
Tosun, et al. 2011 [30]	20	20	<32	<1600	CEN	RCT	6/10	*Some concerns*
Vignochi, et al. 2008 [31,32]	15	14	<32	<1600	CEN	RCT	7/10	*Some concerns*

N: Number. EG: Experimental. CG: Control. wGA: Weeks of gestational age. CEN: Complete enteral nutrition. SN: Similar nutrition between groups. NS: Not specified. RCT: Randomized control trial.

**Table 2:** Characteristics of the physical therapy treatments and their results.

Study	Treatment		Desc.	Age S–I	Period of Aplication	Frequency	Intensity	Who?	Assessments				Outcome Measures	Results	
	E	C							Inicial	During	Final	Follow up		Statistical Significance (*p*)	Group Contrast
Aly, et al. 2004 [25]	PMC + Mas	NA	++	2 postnatal weeks	Until 1.8 kg weight	5 W/S	NS	Mo	Beginning of treatment	NS	1.8 kg weight	NS	ALP	*p* > 0.050	E = C
													PICP	*p* < 0.001	E > C
													Pyd	*p* = 0.984	E = C
													PTH	*p* < 0.001	E > C
													Ca	*p* = 0.002	E > C
Chen, et al. 2010 [33]	PMC	TS	++	1 postnatal week	4 weeks	5 W/S	10 MIN	N	Birth	2nd and 4th postnatal week	6th postnatal week	8th postnatal week	US 2 weeks	*p* = 0.156	E = C
													US 4 weeks	*p* = 0.636	E = C
													US 6 weeks	*p* = 0.031	E > C
													US 8 weeks	*p* = 0.020	E > C
													PICP	*p* > 0.050	E = C
													ALP	*p* > 0.050	E = C
													Weight	*p* > 0.050	E = C
El-Farrash, et al. 2019 [34]	PMC	TS	++	1 postnatal week	4 weeks	5 W/S	10 MIN	NS	Beginning of treatment	NS	End of treatment	NS	DEXA	*p* < 0.001	E > C
													ALP	*p* = 0.005	E < C
													Ca/PO_4_	*p* = 0.040	E < C
													PO_4_	*p* = 0.001	E > C
													CTX	*p* = 0.254	E = C
													Weight	*p* < 0.001	E > C
													Height	*p* > 0.050	E = C
													HC	*p* > 0.050	E = C
Eliakim, et al. 2002 [35]	PMC	TS	++	1 Month	4 weeks	5 W/S	5–10 MIN	NS	1 M	NS	2 M	NS	Leptin	*p* < 0.050	E > C
													IGF-I	*p* < 0.050	E > C
													Weight	*p* < 0.050	E > C
Erdem, et al. 2015 [36]	PMC	UC	++	≤3 postnatal days	4 weeks	5 W/S	5–8 MIN	NS	Beginning of treatment	NS	End of treatment	NS	US	*p* = 0.001	E > C
													Weight	*p* = 0.002	E > C
													Height	*p* = 0.015	E > C
													HC	*p* > 0.050	E = C
Haley, et al. 2012 [37]	TKs	UC	+	32–34 wGA	2 weeks	12 W/S	20 MIN	NS	Beginning of treatment	NS	End of treatment	NS	US	*p* < 0.050	E > C
													Pyd	*p* > 0.050	E = C
													Dpd	*p* > 0.050	E = C
													U-MidOC	*p* < 0.001	E > C
Litmanovitz, et al. 2003 [38]	PMC	TS	++	5–6 days	4 weeks	5 W/S	5 MIN	NS	Beginning of treatment	NS	End of treatment	NS	US	*p* < 0.006	E > C
													BSAP	*p* < 0.050	E > C
													ICTP	*p* < 0.050	E > C
													Weight	*p* < 0.050	E < C
													Height	*p* < 0.050	E > C
													HC	*p* < 0.050	E > C
Litmanovitz, et al. 2016 [39]	PMC	TS	++	<2 postnatal weeks	4 weeks	E1: 10 W/SE2: 5 W/S	10 MIN	NS	Beginning of treatment	2 weeks	End of treatment	NS	US at 2 weeks	*p* < 0.040	E1 > E2 > C
													US at 4 weeks	*p* < 0.030	E1 > C
													Weight	*p* > 0.050	E = C
													Height	*p* > 0.050	E = C
													HC	*p* > 0.050	E = C
Moyer-Mileur, et al. 1995 [11]	PMC	TS	++	NS	4 weeks	6 W/S	5–10 MIN	TO	Beginning of treatment	NS	End of treatment	NS	DEXA	*p* < 0.050	E > C
													ALP	*p* < 0.050	E < C
													PTH	*p* > 0.050	E = C
													Ca (urine)	*p* > 0.050	E = C
													Weight	*p* < 0.050	E > C
													Height	*p* > 0.050	E = C
													HC	*p* > 0.050	E = C
Moyer-Mileur, et al. 2000 [40]	PMC	TS	++	At the beginning of CEN	Until 2 kg weight	6 W/S	5–10 MIN	TO	Beginning of treatment	-	2 kg weight	-	DEXA	*p* < 0.050	E > C
													PICP	*p* = 0.030	E > C
													Pyd	*p* > 0.050	E = C
													Weight	*p* = 0.020	E > C
													Height	*p* > 0.050	E = C
													HC	*p* > 0.050	E = C
Moyer-Mileur, et al. 2008 [26]	PMC	TS	+	31–33 wGA	Until 2 kg weight	6 W/S	5–10 MIN	OT/Mo	Beginning of treatment	-	2 kg weight	-	DEXA	*p* < 0.050	E > C
													BSAP	*p* = 0.040	E > C
													Pyd	*p* > 0.050	E = C
													Weight	*p* = 0.020	E > C
													Height	*p* > 0.050	E = C
													HC	*p* > 0.050	E = C
Nemet, et al. 2002 [27]	PMC	TS	++	32–33 wGA	4 weeks	5 W/S	5–10 MIN	-	Beginning of treatment	-	36–37wGA	-	BSAP	*p* < 0.050	E > C
													PICP	*p* > 0.050	E = C
													ICTP	*p* < 0.050	E < C
													Weight	*p* < 0.050	E > C
Sezer Efe, et al. 2019 [28]	PMC	UC	++	-	30 days	7W/S	7-10 MIN	I	Beginning of treatment	-	End of treatment	-	US	*p* = 0.009	E > C
													Cortisol levels	*p* > 0.050	E = C
													Weight	*p* > 0.050	E = C
													Height	*p* > 0.050	E = C
													HC	*p* > 0.050	E = C
Shaw, et al. 2017 [29]	PMC	UC	++	1 postnatal week	Until 40 wGA	5 W/S	10 MIN	Mo	Beginning of treatment	-	40 wGA	-	US	*p* > 0.050	E = C
													ALP	*p* > 0.050	E = C
													Ca (serum)	*p* > 0.050	E = C
													PO_4_	*p* > 0.050	E = C
													Weight	*p* > 0.050	E = C
													Height	*p* > 0.050	E = C
													HC	*p* > 0.050	E = C
Tosun, et al. 2011 [30]	PMC	UC	++	NS	4 weeks	5 W/S	5–10 MIN	NS	Beginning of treatment	NS	End of treatment	NS	US	*p* < 0.050	E > C
													Weight	*p* > 0.050	E = C
													Height	*p* > 0.050	E = C
													HC	*p* > 0.050	E = C
Vignochi, et al. 2008 [31,32]	PMC	UC	++	32–33 wGA	Until HD/2 kg weight	5 W/S	15 MIN	NS	Beginning of treatment	NS	HD	NS	DEXA	*p* < 0.050	E > C
													Weight	*p* < 0.001	E > C
													Height	*p* > 0.050	E = C
													HC	*p* > 0.050	E = C
													BSAP	*p* < 0.001	E > C
													Dpd	*p* < 0.003	E < C
													Ca (serum)	*p* > 0.050	E = C
													PO_4_	*p* > 0.050	E = C
													PTH	*p* > 0.050	E = C

S-I: Start intervention. E: Experimental. C: Control. PMC: Passive mobilizations with gentle joint compression. Mas: Massage. TKs: Tactile kinesthetic stimulation. TS: Tactile stimulation. UC: Usual care. Desc.: Level of description of the treatment. ++: Specifically described. +: Little described. NS: Not specified. CEN: Complete enteral nutrition. wGA: Weeks of gestational age. HD: Hospital discharge. W/S: Weekly sessions. MIN: Minutes. Mo: Mother. N: Nurse. OT: Occupational therapist. M: Months of age. HD: Hospital discharge. Serum biomarkers: PICP, PTH, Leptin, IGF-I, BSAP, ICTP, ALP, Ca PO_4_, cortisol levels. Urine biomarkers: Pyd, Dpd, Ca/PO_4_, CTX, U-MidOC, Ca. US: Ultrasound. DEXA: Densitometry. HC: Head circumference E = C: No differences between groups. E < C: Control higher. E > C: Experimental Higher.

**Table 3:** Methodological quality assessed with PEDro scale.

PEDro Scale	1	2	3	4	5	6	7	8	9	10	11	T
Aly, et al. 2004 [25]	X	X	X							X		3
Chen, et al. 2010 [33]	X	X		X				X		X	X	5
El-Farrash, et al. 2019 [34]	X	X	X	X			X			X	X	6
Eliakim, et al. 2002 [35]		X		X						X	X	4
Erdem, et al. 2015 [36]	X	X		X	X		X	X		X	X	7
Haley, et al. 2012 [37]	X	X	X	X	X		X			X	X	7
Litmanovitz, et al. 2003 [38]	X	X		X			X	X		X	X	6
Litmanovitz, et al. 2016 [39]	X	X	X	X			X			X	X	6
Moyer-Mileur, et al. 1995 [11]	X	X		X						X	X	4
Moyer-Mileur, et al. 2000 [40]	X	X	X	X	X		X	X		X	X	8
Moyer-Mileur, et al. 2008 [26]	X	X	X	X			X			X	X	6
Nemet, et al. 2002 [27]	X	X		X	X			X		X	X	6
Sezer Efe, et al. 2019 [28]	X	X	X	X	X		X	X		X	X	8
Shaw, et al. 2017 [29]	X	X	X	X			X	X		X	X	7
Tosun, et al. 2011 [30]	X	X		X			X	X		X	X	6
Vignochi, et al. 2008 [31,32]	X	X	X	X			X	X		X	X	7

(1) Eligibility criteria were specified. (2) Random allocation. (3) Allocation was concealed. (4) The groups were similar at baseline. (5) There was blinding of all subjects. (6) There was blinding of all therapists. (7) There was blinding of all assessors. (8) Measures of at least one key outcome were obtained from more than 85% of the subjects initially allocated to groups. (9) All subjects for whom outcome measures were available received the treatment or control condition as allocated or where this was not the case, data for at least one key outcome was analyzed by “intention to treat”. (10) The results of between-group statistical comparisons are reported for at least one key outcome. (11) The study provides both point measures and measures of variability for at least one key outcome. T = Total score. X: Meets the criteria.

**Table 4:** Risk of bias assessed with the ROB-2 Cochrane scale.

Study	Randomization Process	Deviations from Intended Interventions	Missing Outcome Data	Measurement of the Outcome	Selection of the Reported Result	Overall
Aly, et al. 2004 [25]	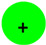	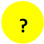	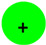	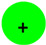	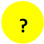	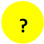
Chen, et al. 2010 [33]	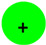	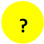	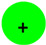	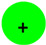	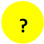	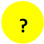
El-Farrash, et al. 2019 [34]	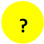	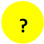	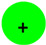	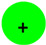	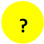	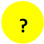
Eliakim, et al. 2002 [35]	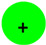	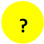	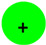	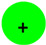	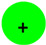	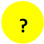
Erdem, et al. 2015 [36]	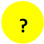	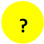	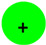	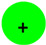	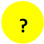	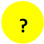
Haley, et al. 2012 [37]	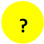	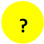	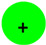	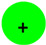	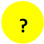	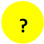
Litmanovitz, et al. 2003 [38]	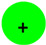	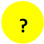	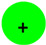	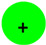	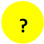	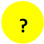
Litmanovitz, et al. 2016 [39]	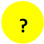	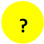	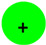	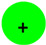	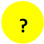	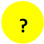
Moyer-Mileur, et al. 1995 [11]	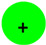	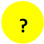	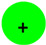	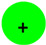	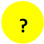	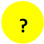
Moyer-Mileur, et al. 2000 [40]	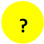	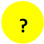	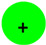	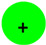	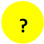	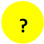
Moyer-Mileur, et al. 2008 [26]	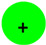	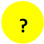	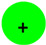	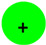	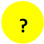	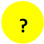
Nemet, et al. 2002 [27]	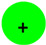	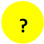	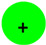	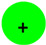	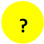	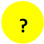
Sezer Efe, et al. 2019 [28]	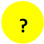	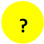	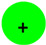	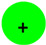	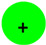	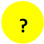
Shaw, et al. 2017 [29]	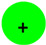	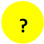	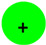	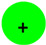	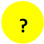	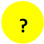
Tosun, et al. 2011 [30]	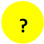	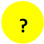	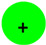	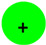	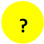	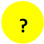
Vignochi, et al. 2008 [31,32]	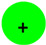	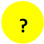	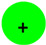	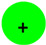	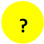	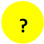

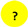
: Some concerns; 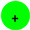: Some concerns; 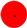: High Risk.

## Data Availability

The data that support the findings of this study are available from the corresponding author, (G.T.-F.), upon reasonable request.
